# The diagnostic evaluation of dual-source CT (DSCT) in the diagnosis of coronary artery stenoses

**DOI:** 10.12669/pjms.291.2645

**Published:** 2013

**Authors:** Ziqiao Lei, Jin Gu, Qing Fu, Heshui Shi, Haibo Xu, Ping Han, Jianming Yu

**Affiliations:** 1 Ziqiao Lei, Department of Radiology, Union Hospital, Wuhan, China.; 2 Jin Gu, Department of Radiology, Union Hospital, Wuhan, China.; 3 Qing Fu, Department of Radiology, Union Hospital, Wuhan, China.; 4 Heshui Shi, Department of Radiology, Union Hospital, Wuhan, China.; 5 Haibo Xu, Department of Radiology, Union Hospital, Wuhan, China.; 6 Ping Han, Department of Radiology, Union Hospital, Wuhan, China.; 7 Jianming Yu, Department of Radiology, Union Hospital, Wuhan, China.

**Keywords:** Coronary artery stenoses, Dual-source CT, Computed tomography, Coronary angiography

## Abstract

***Objective:*** To evaluate the diagnostic accuracy of dual-source computed tomography (DSCT) in the diagnosis of coronary artery stenoses by comparing with conventional coronary angiography (CCA).

***Methodology:*** CCA and DSCT were performed in 64 patients with suspected coronary artery disease (CAD) respectively (46 male, 18 female, age from 48 to 82 years old, mean 68.18 years). Various post-processing reconstructions of coronary artery and its branches, such as volumetric imaging, multi-planar reconstruction, curved planar reconstruction, maximum intensity projection were used. The coronary segments, with statistical evaluations combined with its diameter ≥1.5mm were collected to analyze the diagnosis accuracy of DSCT on coronary artery stenoses, with CCA as the gold standard.

***Results:*** About 4.14% of coronary artery segments could not be evaluated, while 95.86% were evaluable arteries, the sensitivity, specificity, positive and negative predictive value of DSCT for detecting coronary artery stenoses were, 93.58%, 99.61%, 95.31% and 99.48% respectively. There were no significant differences in the diagnostic accuracy of coronary artery stenoses between DSCT and CCA.

***Conclusion:*** DSCT is a reliable tool that is accurately appropriate for patients with CAD, as it has a higher accuracy and specificity, which is valuable in the screening of CAD.

## Introduction

 Multi-slice computed tomography (MSCT) has gained a great acceptance in clinical application as a reliable and non-invasive method to detect coronary heart disease.^1^^, ^^2^ Dual-source computed tomography (DSCT) is one of the latest innovations in multi-slice CT technology, which is composed of two x-ray tubes and two corresponding detectors. The gantry rotation time is 0.33s, the temporal resolution of 83ms is faster than the other MSCT before. It has a great display of coronary artery and the evaluation of coronary artery stenoses with diameter more than 1.5mm.^3^^-^^5^

 We retrospectively evaluated subjects who prospectively underwent SCA and DSCT in order to evaluate the application value of DSCT in patients with coronary artery stenoses as well as systematic analysis of its value in the diagnosis of varying coronary artery stenoses.

## Methodology

***Patients: ***From December 2008 until April 2011 we included 64 patients who were referred to our hospital for DSCT (Somatom Definition, Siemens Medical Solutions, Forchheim, Germany) for suspected coronary artery disease (CAD), Exclusion criteria were: unstable angina, allergic to iodinated contrast material, renal insufficiency, severe respiratory function impairment and heart failure. 

## Methods

***DSCT data acquisition: ***All the patients cooperated with the DSCT examination at the time of scan, without taking any anti-arrhythmic drugs. The heart rate ranged from 58 to 96beats/min, and averaged 70.3beats/min. We used artificial intelligence trigger system (bolus tracking mode) and the scan was started with a delay time of 5s after the density in the aortic root exceeded a density value of 100HU. We used an injector (Medrad Stellant) with high-pressure syringe of 3.8-4.5 ml/s rate of 65-75ml of the contrast medium injection, then a saline flush followed (50ml at 4.0ml/s of 0.9%). The coronary angiography was performed with DSCT at 0.6mm collimation, 120kVp.330ms gantry rotation time, automatic pitch or manual one (0.2 to 0.44), 0.75 reconstruction slice thickness. During the scan, patients were in deep inspiration breath-hold status with a scan range from tracheal subcarinal to diaphragmatic dome in a craniocaudal direction. ECG was recorded simultaneously in order to make image reconstruction.

***DSCT image post-processing: ***The images were transferred to separate workstations for post-processing. The initial axial images reconstruction was set with the reconstruction window starting at 75% (equivalent to end of diastolic phase) and 45% (equivalent to end of systolic phase) of the cardiac cycle. The images were reconstructed with a section thickness of 1mm and an increment of 0.5mm by using a medium smooth reconstruction kernel (B35f). Then some post-processing work was analyzed such as MPR (multi-planar reformation), CPR (curved planar reformation), MIP (maximum intensity projection), VRT (volume rendering techniques) and optimized analysis software for coronary vessels. VRT was used to observe three dimensional structures of coronary artery while MPR, CPR and MIP were used to manifest coronary artery wall, the lumen changes and the relationship with the adjacent vascular and other organizations.

***Coronary artery section and evaluation standard: ***The American College of Cardiology (ACC) and the American Heart Association (AHA) classification of coronary segments were adopted as guidelines and basis for imaging evaluation.^6^ Right coronary artery (RCA) is divided into proximal, middle and distal segments (otherwise the right atrium branch, right ventricle branch, right acute marginal branch, posterior interventricular branch and so on). The left coronary artery is divided into left main coronary artery (LM) and left circumflex branch (LCX). LAD is divided into proximal, middle and distal segments, LCX into proximal and distal segments (otherwise the interval branch, the first, second, third diagonal branches, the first, second left obtuse marginal branch). All the coronary segments were evaluated gradually in segmentation if the vessel diameter ≥1.5mm. The evaluation was possible even with a few artifacts but still met the demands of the diagnosis. The vessel was not evaluated if any segment of the coronary main branch could not be evaluated.

***C*** ***CA examination: ***CCA was successfully performed in all 64 patients before or after DSCT, 3-28 days interval between the two examinations, mean 16 days. Various perspectives were applied to observe the coronary artery, at least four perspectives to left coronary artery and two to right coronary artery.

***Assessment methods: ***Double-blind analysis between two independent investigators of DSCT and SCA output were compared. For coronary vessel diameter ≥1.5mm, analysis of coronary artery stenoses was done with the use of internationally accepted method of visual diameter, calculated as follows:

Degree of stenosis = (normal vessel diameter proximal to stenosis-diameter of stenosed part)/ normal vessel diameter proximal to stenosis x 100%.

Grade of coronary stenosis^7^: mild <50% diameter stenosis, moderate ≥ 50% to <75%, severe ≥ 75% and vascular occlusion 100%.

***Statistical analysis: ***Statistical analysis was done using SPSS 15.0 software. Comparing with the CCA results, we calculated the sensitivity, specificity, positive and negative predictive value of the DSCT coronary artery pathological lesions; Paired χ2 test was used to detect the differences between DSCT and SCA in their ability to manifest coronary artery lesions. For all the data, P values of ≤ 0.05 were considered to be significant. 

## Results

**Fig.1:**
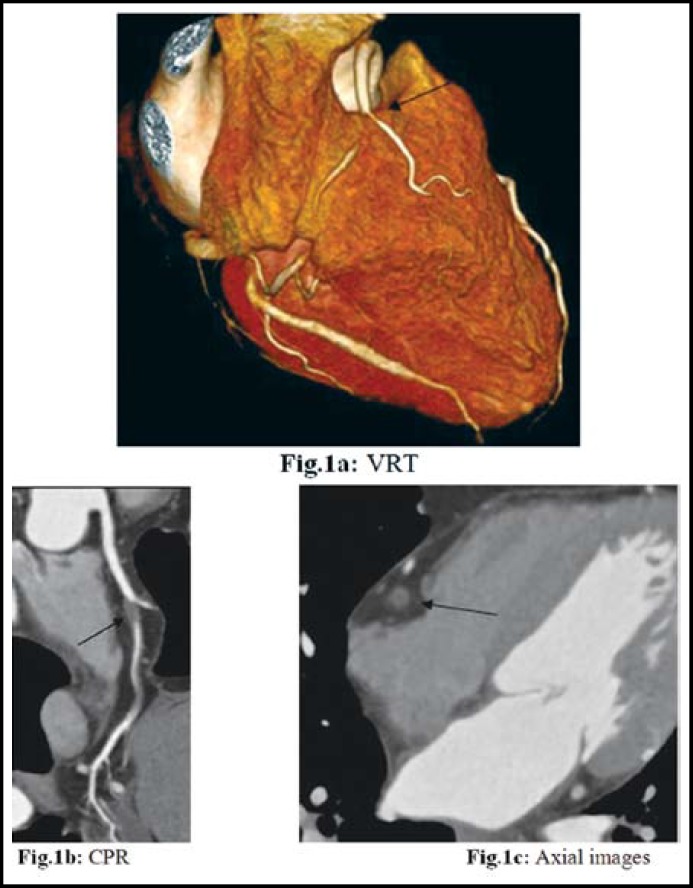
The right coronary showing a middle occluded right coronary artery as well as a distal severe stenosis

**Fig.2:**
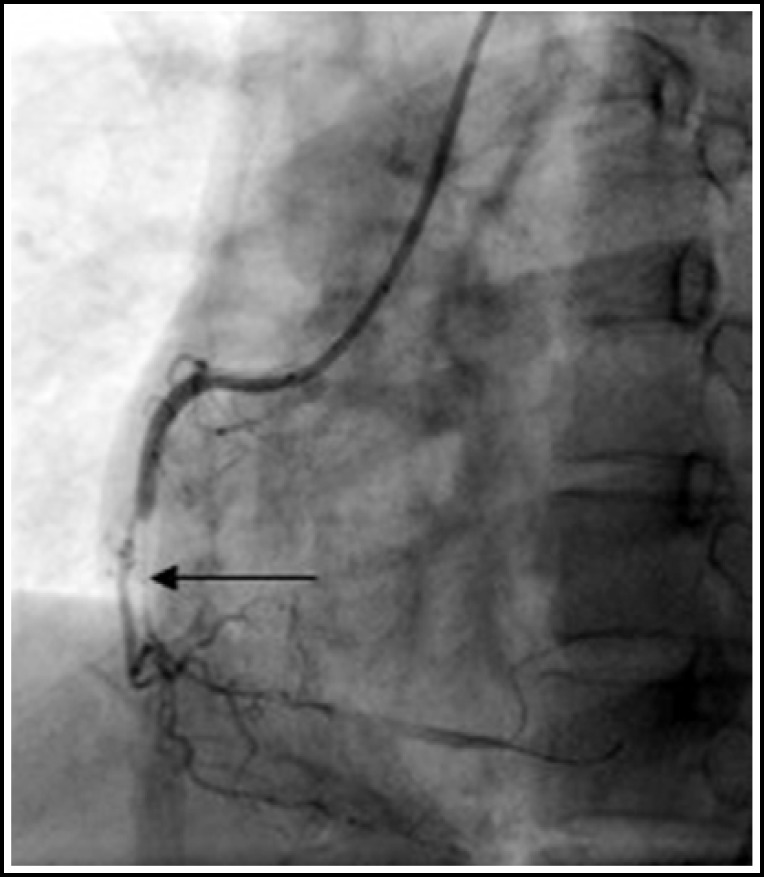
Invasive coronary angiogram of the right coronary artery showing a middle and distal severe stenosis

 Sixty four patients successfully underwent the DSCT and SCA examinations (46 men, 18 women, aged 48-82 years, mean age 61 years). There were 845 segments in SCA and 810 segments in DSCT of the coronary artery that could be assessed with the diameters>1.5mm, while, 4.14% (35/845) segments could not be evaluated separately for following reasons: 19 segments of motion artifact, 12 segments of dense calcification and 4 segments of negative lumen manifestation. These cases were excluded from our study. In the 36 segments of mild stenosis which was manifested in SCA, 29 segments were correctly diagnosed, 3 segments were misdiagnosed and 4 segments were overestimated (3 was moderate stenoses and 1 was severe) in DSCT. In the 30 segments of moderate, diagnosis which is manifested in SCA, 26 segments were correctly diagnosed (Fig.1-2), 3 segments were miss diagnosed, and one segment was overestimated in DSCT. In the 65 segments of severe stenoses which was manifested in SCA, 61 segments were correctly diagnosed, 4 segments were misdiagnosed and 3 segments were overestimated in DSCT (Table-I). According to the results mentioned above, the sensitivity, specificity, positive and negative predictive value of the DSCT evaluation for the severe coronary artery stenoses were 93.85%, 99.61%, 95.31%, 99.48% respectively. Paired χ2-test found P value >0.05, showing no statistically significant difference between DSCT and CCA on manifestation of severe coronary artery stenosis.

## Discussion

 As one of motives of the development of multi-slice CT technology, non-invasive cardiac imaging has been limited by low resolution. Therefore, the imaging results of the 16-slice or 64-slice CT still have some discordance with CCA.^8^ DSCT coronary angiography has more advantages over SCA. DSCA has dual X-ray tubes, a gantry rotation time of 0.33s thus resulting in a temporal resolution of 82.5ms. The 90° rotation can generate excellently higher imaging quality and scan speed compared to single-source CT, and a significant improvement of time resolution and clinical application as well as patient comfort. Secondly, DSCT possesses higher spatial resolution, which combines with the spatial resolution of <0.4mm that can clearly demonstrate 2 to 3 grade branches of coronary artery with diameter ≥1.5mm, making the detection rate increased substantially to display accurate small plaques or calcification of the coronary artery.^9^^,^^10^

a.VRT       b.CPR       c.Axial images

 Several international studies suggest that DSCT has a good consistency than SCA in the diagnosis of CAD. DSCT can display most of coronary arteries and could accurately assess the degree of stenosis.^10^ By comparing the results of DSCT with that of SCA, it was found that DSCT can clearly show coronary distribution and 2 to 3 grade branches of coronary artery with diameter ≥1.5mm. The diameter ≥1.5mm of coronary artery was chosen as an evaluation object because arteries of this much size can be revascularized.^11^

 In this study, DSCT image has a good quality rate of 95.86%, and the sensitivity, specificity, positive and negative predictive value of coronary artery stenoses at severe levels. DSCT has a higher reliability and diagnostic value on severe coronary artery stenoses. It has a high sensitivity of 93.8%, a specificity of 99.61%, 95.31% positive predictive value and negative predictive value of 99.48%. The results are in accordance with that of Scheffel et al^12^ research output whereby 30 cases of CAD patients underwent DSCT coronary angiography without controlling heart rate, the sensitivity, specificity, positive and negative predictive value of coronary artery segmental stenoses are 96.4%, 85.7%, 85.7 and 99.4%.

 Heart rate control is not mandatory prior to the DSCT scan. Johnson et al^8^ concluded that DSCT coronary angiography can provide high quality diagnostic value of coronary arteries, heart valves and heart imaging after re-evaluation of them on 24 patients with 44-92 beats/min heart rate. Scheffel et al^12^ study and Achenbanh et al^10^ study all came to the similar conclusions. In our study, Patient's heart rate before the test was 58 to 96 / min, an average of 70.3 / min without taking β- blockers or other drugs to reduce heart rate. While the single source 64-slice CT requires the patients heart rate to be maintained at 60 to 65 beats/min.^13^

**Table-I:** DSCT analysis of 64 patients

*Comparative indicators*	*Mild*	*Moderate*	*Severe*
True positive (segments)	29	26	61
True negative (segments)	679	731	764
False positives (segments)	4	1	3
False negative (segments)	3	3	4
Sensitivity [n (%)]	29/32(90.63)	26/29(89.66)	61/65(93.85)
Specificity [n (%)]	679/683(99.41)	731/732(99.86)	764/767(99.61)
Positive predictive value [n (%)]	29/33(87.88)	26/27(96.30)	61/64(95.31)
Negative predictive value [n (%)]	679/682(99.56)	731/734(99.59)	764/768(99.48)

 The study found that DSCT for coronary artery stenoses had a high negative predictive value is important to rule out coronary artery lesions. This means that invasive examination of CCA could be avoided for the patients with high negative predictive value in DSCT. However, positive predictive value of mild stenoses is low.

 Nineteen segments of cardiac motion artifacts, 12 segments of dense calcification and 4 segments of poor lumen enhancement were analyzed retrospectively. The cardiac motion artifacts were caused by arrhythmia (2 patients with atrial fibrillation, one patient with sinus arrhythmia), indicating that although there are no significant requirements on heart rate speed, an irregular cardiac rhythm is the key restrictions. When the coronary walls are calcified, it is difficult to accurately determine the degree of stenosis or it could be overestimated because of the partial volume effects in DSCT. Diffuse mixed calcified coronary artery plaques are often accompanied by vascular positive remodeling and no significant stenoses of the artery lumen, so the DSCT examination shows overestimation of luminal stenoses, the accuracy of coronary artery angiography results on both DSCT and single source 64-slice CT are affected by coronary artery calcification.^14^ It is difficult to distinguish the vessel lumen with density extensive calcification, leading to an incorrect evaluation of the degree of luminal stenosis.^15^^-^^17^ Several studies have shown that the sensitivity, specificity, positive and negative predictive value of 64-slice CT in detecting severe calcification (score>400) are 93%, 67%, 93% and 67% respectively.^13^

 In Scheffel et al^12^ study on DSCT the sensitivity, specificity, positive and negative predictive values are 96.1%, 94.8%, 86% and 98.7% respectively which are better than the 64-slice CT. Although diagnostic accuracy of DSCT is affected by severe calcification, it has been greatly improved in comparison to other non-invasive coronary artery examination methods. Poor lumen enhancement has been correlated with the amount of contrast agent, scan delay time and the degree of proximal segment coronary artery stenoses. The 4 segments in the study which couldn’t be evaluated were caused by proximal coronary artery stenoses.

 A total of 8 stents in the group were assessed. DSCT coronary angiography can affirm a clear indication to display the stent lumen and plaques at both sides. DSCT and CCA examination have the same capability to check for re-stenosis detection and classification of diagnosis, which suggests that DSCT has a high accuracy in the diagnosis of stent re-stenosis, and it is the same with reports of Das et al.^18^

 In short, DSCT coronary angiography has solved problems such as time resolution.^9^^,^^10^ We can get better image quality without the need for patients to heart rate control. DSCT for noncalcified coronary arteries show a higher reliability and diagnostic value. The sensitivity, specificity, positive and negative predictive values, for evaluating the degree of stenosis by DSCT, all meet the clinical requirements. So DSCT can be used as the preferred method in preoperative screening for CCA in patients with clinical suspicion of CAD or postoperative coronary stent implantation or patients on follow-up after coronary artery bypass grafting surgery.

## References

[1] Dodd JD, Rieber J, Pomerantsev E (2008). Quantification of nonculprit coronary lesions: comparison of cardiac 64-MDCT and invasive coronary angiography. AJR Am J Roentgenol.

[2] Ropers D, Rixe J, Anders K (2006). Usefulness of multidetector row spiral computed tomography with 64- x 0.6-mm collimation and 330-ms rotation for the noninvasive detection of significant coronary artery stenoses. Am J Cardiol.

[3] Lell MM, Panknin C, Saleh R (2007). Evaluation of coronary stents and stenoses at different heart rates with dual-source spiral CT (DSCT). Invest Radiol.

[4] Brodoefel H, Burgstahler C, Tsiflikas I (2008). Dual-source CT: effect of heart rate, heart rate variability, and calcification on image quality and diagnostic accuracy. Radiology.

[5] Ropers U, Ropers D, Pflederer T (2007). Influence of heart rate on the diagnostic accuracy of dual-source computed tomography coronary angiography. J Am Coll Cardiol.

[6] Scanlon PJ, Faxon DP, Audet AM (1999). ACC/AHA guidelines for coronary angiography A report of the American College of Cardiology/American Heart Association Task Force on practice guidelines (Committee on Coronary Angiography) Developed in collaboration with the Society for Cardiac Angiography and Interventions. J Am Coll Cardiol.

[7] Nieman K, Oudkerk M, Rensing BJ (2001). Coronary angiography with multi-slice computed tomography. Lancet.

[8] Johnson TR, Nikolaou K, Wintersperger BJ (2006). Dual-source CT cardiac imaging: initial experience. Eur Radiol.

[9] Flohr TG, McCollough CH, Bruder H (2006). First performance evaluation of a dual-source CT (DSCT) system. Eur Radiol.

[10] Achenbach S, Ropers D, Kuettner A (2006). Contrast-enhanced coronary artery visualization by dual-source computed tomography-initial experience. Eur J Radiol.

[11] Ropers D, Baum U, Pohle K (2003). Detection of coronary artery stenoses with thin-slice multi-detector row spiral computed tomography and multiplanar reconstruction. Circulation.

[12] Scheffel H, Alkadhi H, Plass A (2006). Accuracy of dual-source CT coronary angiography: First experience in a high pre-test probability population without heart rate control. Eur Radiol.

[13] Leschka S, Alkadhi H, Plass A (2005). Accuracy of MSCT coronary angiography with 64-slice technology: first experience. Eur Heart J.

[14] Raff GL, Gallagher MJ, O’Neill WW, Goldstein JA (2005). Diagnostic accuracy of noninvasive coronary angiography using 64-slice spiral computed tomography. J Am Coll Cardiol.

[15] Zheng MW, Li JY (2009). Dual-source computed tomographic coronary angiography. Clinical Invest.

[16] Dev D, Lee CJ, Ohba M (2008). Image quality and artifacts in coronary CT angiography with dual-source CT: initial experience. J Cardiovasc Computed Tomogr.

[17] Rajab MI, Eskandar AA (2011). Enhancement of radiographic images in patients with lung nodules. Thoracic Cancer.

[18] Das KM, El-Menyar AA, Salam AM (2007). Contrast-enhanced 64-section coronary multidetector CT angiography versus conventional coronary angiography for stent assessment. Radiology.

